# Cooperation, social norm internalization, and hierarchical societies

**DOI:** 10.1038/s41598-020-71664-w

**Published:** 2020-09-21

**Authors:** Pablo Lozano, Sergey Gavrilets, Angel Sánchez

**Affiliations:** 1 Grupo Interdisciplinar de Sistemas Complejos, Departamento de Matemáticas, Universidad Carlos III de Madrid, 28911 Leganés, Madrid, Spain; 2 Unidad Mixta Interdisciplinar de Comportamiento y Complejidad Social (UMICCS), Madrid, Spain; 3 Department of Ecology and Evolutionary Biology, Department of Mathematics, National Institute for Mathematical and Biological Synthesis, Center for the Dynamics of Social Complexity, University of Tennessee, Knoxville, TN 37996 USA; 4 Institute for Biocomputation and Physics of Complex Systems (BIFI), University of Zaragoza, 50018 Zaragoza, Spain; 5 UC3M-Santander Big Data Institute (IBiDat), Universidad Carlos III de Madrid, 28903 Getafe, Madrid, Spain

**Keywords:** Computational science, Statistical physics, Biological physics

## Abstract

Many animal and human societies exhibit hierarchical structures with different degrees of steepness. Some of these societies also show cooperative behavior, where cooperation means working together for a common benefit. However, there is an increasing evidence that rigidly enforced hierarchies lead to a decrease of cooperation in both human and non-human primates. In this work, we address this issue by means of an evolutionary agent-based model that incorporates fights as social interactions governing a dynamic ranking, communal work to produce a public good, and norm internalization, i.e. a process where acting according to a norm becomes a goal in itself. Our model also includes the perception of how much the individual is going to retain from her cooperative behavior in future interactions. The predictions of the model resemble the principal characteristics of human societies. When ranking is unconstrained, we observe a high concentration of agents in low scores, while a few ones climb up the social hierarchy and exploit the rest, with no norm internalization. If ranking is constrained, thus leading to bounded score differences between agents, individual positions in the ranking change more, and the typical structure shows a division of the society in upper and lower classes. In this case, we observe that there is a significant degree of norm internalization, supporting large fractions of the population cooperating in spite of the rank differences. Our main results are robust with respect to the model parameters and to the type of rank constraint. We thus provide a mechanism that can explain how hierarchy arises in initially egalitarian societies while keeping a large degree of cooperation.

## Introduction

In nature, animals often benefit from living in groups due to reduced predation risk and increased availability of mates. At the same time, group living can result in strong competition for critical resources. When unfamiliar individuals find themselves in a group, they can compete violently initially, but then their competition can subdue once stable dominance-subordinate relationships form. Dominance hierarchies are found in many different social animals, including hens, cows, fish, and crabs, rats, primates and insects, especially wasps and bumblebees^1–9^. Among different dominance patterns, the most typical structure is a linear hierarchy known as a classical pecking order^10,11^. Linear hierarchies are formed both in nature and laboratory in a vast range of species, including humans. Two main hypotheses have been proposed to explain linear hierarchies: they arise from differences in the intrinsic attributes of animals, or they result from the dynamics of social interaction^1,12–14^. Several mathematical models^15–21^, suggest that dominance orders in animal societies could indeed result from a self-organizing process through a double reinforcement mechanism: winners increase their probability of winning, and losers theirs of losing. In addition, some agent-based models^22,23^ have shown that as the intensity of aggression increases, the double reinforcement mechanism may cause an egalitarian society to change into a despotic one. Relevant works in this context also include the papers by Ben-Naim and Redner^24,25^ and by Bonabeau et al.^15,26,27^ to which we will come back below.

Social structure, whether it results from dominance interactions or from any other mechanism, has a strong impact on the cooperative behavior of individuals^28–37^. For our present purposes, we understand cooperation as working together to achieve a common goal or obtain some benefit^37,38^. Note that we do not restrict ourselves to social dilemma situations, in so far as working together might be more beneficial than doing it alone, and our results are therefore quite general. In the case of nonhuman primates, the characteristics of dominance hierarchies strongly affect cooperative outcomes, with steep hierarchies generally reducing cooperation. For example, in chimpanzees, known to live in basically linear hierarchies, cooperation is weak^33,39^ while species with more relaxed hierarchies, such as cottontop tamarins, are much more cooperative^32,33,35^. Much less is known about the interplay of hierarchical structure and cooperative behavior in humans, but there is an experimental evidence that steep hierarchies also result in lower cooperation^40^ and that less steep hierarchies preserve it^41^. Other theoretical analyses have shown that dominant individuals (e.g., leaders with higher motivation or strength) can act seemingly altruistically in between-group conflicts—expending more effort and having lower reproductive success—than their subordinate group-mates^42–45^. In this context, an open question is how can large-scale cooperative societies arise if a strong hierarchical structure is present and sustained by physical or economic interactions.

A crucial point in the discussion above is the fact that human behavior is more complex than animal behavior. Part of this complexity is that human behavior is affected by norms and values that are transmitted culturally. A social norm is a set of behaviors that one is expected to follow in a specific context, and expects others to follow in a given social situation^46,47^. Social norms can play a fundamental role to coordinate aspects of social behavior, but they arise and evolve as a result of individual behaviors, expectations, and interactions with others^48,49^. Thus, humans learn the expected behavior for each specific context—which means they act according to prevalent norms—from sources such as family, via education and religion, information sources including media or books, and from interaction with and observation of others. In some cases, norms become internalized: they are then an end in themselves instead of a tool to simplify tasks or help in choosing the proper behavior to avoid social punishment. This internalization process is reminiscent of imitation and imprinting, that has been observed, e.g., in species of birds and mammals^50^. Adherence to the associated norms and the corresponding behavior are subsequently reinforced by approval from others, rewards, and punishment when one fails to follow the norm.

In this paper, we study a model with two stages: first individuals attempt to solve a collective action problem and then they engage in dyadic conflicts over shares of the collectively produced resource. These dyadic fights, in turn, modify the dominance rank of the involved individuals and, as a consequence, their future decisions. Norm internalization is included in the picture through a function that individuals optimize when choosing their behavior, and evolves through differential reproduction according to payoffs received. Our model can be understood as a combination of the ideas on norm internalization and collective action in Refs.^48,49^ with the double reinforcement mechanism in Ref.^25^ As we discuss in detail below, we have found that the interplay of these two features gives rise to cooperative, hierarchical societies starting from an egalitarian situation.

**Table 1:** Variables, parameters and attributes of the model and their meanings.

*n*	Group size
*G*	Number of groups
$S=nG$	Number of individuals in the society
*T*	Number of rounds the whole society lasts
*Q*	Rounds that every generation lasts
*e*	Error rate of optimization
*v*	How much the cooperation is valued in the utility function
$x_i$	Dichotomous variable: *i*th individual’s cooperation in collective action
$r_i$	Discrete variable: *i*th individual’s score
$\eta _i$	Ability of the *i*th individual to internalize the norm
$\mu$	Probability to revise the strategy
$\pi _{CA}$	Resource from the collective action
$\pi ^*$	Accumulated resources
$c_{opt}$	Cost of optimizing the payoffs in the utility function
$c_{int}$	Cost of internalizing the norm in the utility function

## Model

### Model setup

Our model considers of *S* individuals living in groups of constant size *n* (a summary of the model parameters and attributes can be found in Table 1). Each individual can interact only with other individuals in her group. Generations are discrete and non-overlapping, and consist of *Q* rounds with three stages: collective action, fights over resources, and strategy revision. Reproduction takes place at the end of each generation. Every individual has a series of attributes that can evolve over time:

- An attribute which measures the score (position in the social scale): $r_k\in {\mathbb {Z}}$.
- A binary attribute $x_i$, 0 or 1, indicating whether the individual cooperates or not in the collective action.
- An attribute which measures norm internalization: $\eta \in {\mathbb {R}}$, with $0 \le \eta \le 1$, where the norm is to cooperate in the collective action. Let us note that this attribute is a property of the individual, and that in general it differs from one individual to the next. In contrast with the previous two parameters, this one does not change during the evolution of an individual, being modified only at the reproduction phase as discussed below.

At the beginning of the simulations, the binary attribute *x*, describing the first decision of the individual, is randomly selected 0 or 1 with a $50\%$ probability. Hence, the mean value for the cooperation at the beginning of each round is 0.5. The initial values for $\eta$, the sensitivity to the norm, follow a uniform random distribution between 0 and 0.05, implying very little norm internalization at the beginning of the simulation. Scores are all set to 0 at the beginning of the simulation, so all individuals are equal. Let us know go in detail through the steps of the model dynamics.

### Evolution

**Figure 1:**
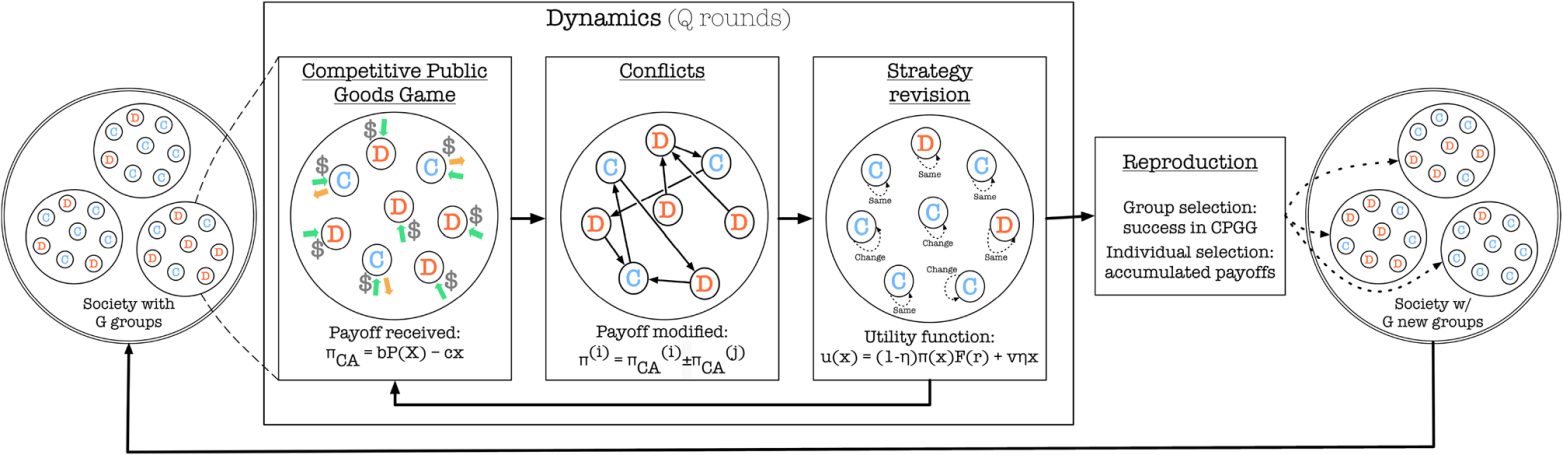
Model flow.

Model evolution consists of collective action, fights, computation of the utility function for the individuals, subsequent revision of strategies, computation of fitness and reproduction (see Fig. 1).

#### Collective action

In this stage, each individual cooperates or not in the collective action to obtain a resource. When cooperating ($x_i=1$), each individual makes an effort spending an amount *c* of accumulated payoffs. The aggregated resource obtained from all the individuals’ efforts is multiplied by a factor *b* and then shared equally among all group members, irrespective of whether they have collaborated or not. The payoff from the collective action for each individual *k* is calculated as

$$\begin{aligned} \pi ^{(k)}_{CA}=bP(X)-cx. \end{aligned}$$

The function *P*(*X*) gives the normalized value of the resource: $P(X)=X/{\bar{X}}$, where *X* is the total effort of the group to which the individual belongs to, and ${\bar{X}}$ the mean effort of cooperation over all groups, while *b* is the multiplier of the collective effort and *c* is the cost of cooperating. This is the first ingredient of the model we take from Ref.^48^.

#### Fights

In this second stage, dyadic conflicts may occur among randomly chosen individuals from the same group. In these conflicts, the contestants attempt to take each others’ share of the resource. We assume that the individual whose score is higher wins the fight 90% of the times, otherwise she loses. While this may seem a very extreme choice, we stress that we have tried other possibilities, including making the probability of a win dependent on the rank difference with very similar results. We decided to keep this simple choice for computational speed and convenience. For every individual and every round, we pick an opponent at random from the rest of the group the agent belongs to. In this manner, on average every agent is in two fights per rounds, is in at least one fight, and can be in up to $n-1$ fights. The winner increases her score by one unit and takes the loser’s last payoff from the collective action $\left( \pi ^{(\text {winner})}_{CA}=\pi ^{(\text {winner})}_{CA}+\pi ^{(\text {loser})}_{CA}\right)$, while the loser’s score decreases by one unit and she loses her last payoff $\left( \pi ^{(\text {loser})}_{CA}=0\right)$. Payoffs after every round are accumulated, so each agent has an amount of resource $\pi ^*$, which accounts for all the payoffs that the agent manages to keep along with her history. In this manner, $\pi _{CA}$ is used in the strategy update process, and $\pi ^*$ is taken into account in the evolutionary process afterward. Note that there is no hierarchy between groups: groups provide a setup for collective action and for an additional component of fitness via group selection.

#### Utility function and norm internalization

We introduce a continuous norm internalization attribute, $\eta$, genetically controlled by a single locus with a continuum of alleles^48^. The attribute $\eta$ is constant over the individual’s life and changes only through random mutations during reproduction. With this attribute, the utility function $u_\eta$ for the *i*th individual is computed as

$$\begin{aligned} u_\eta ^{(i)}(x_i)=(1-\eta _i)F(r_i)\pi ^{(i)}+v\eta _ix_i, \end{aligned}$$

where $\pi ^{(i)}$ is the expected payoff for the *i*th individual, computed via myopic optimization. The utility function thus has two components: a purely material contribution, the first term in the equation above, and another one that arises from the satisfaction of the individual with her behavior through a norm internalization process. When $\eta =0$, the individual is undersocialized and only wants to maximize her payoff from the collective action, not caring about the norm. If $0<\eta <1$, following the norm will be a part of the individual’s preference, contributing to the individual’s utility along with the payoff. Individuals with $\eta =1$ will be oversocialized, do not care about their material payoff, their utility arising simply from following the norm. The parameter *v* defines the maximum value of cooperating. So far, this is the same as the utility function of Ref.^48^. For the purposes of the present paper, we introduce an additional, conflict-related ingredient, namely a function *F*. Specifically, we assume that individuals are aware of their rank in the group and about the possibility of future fights. In this case, low-rank individuals are expected to discount their future resource because they might lose it to stronger individuals. That is, we assume that individuals are capable of certain foresight^44,45^. This is captured in a heuristic way by function $F(r)=1/(1+\exp (-\gamma r))$, where *r* is the score of the individual and $\gamma$ is a parameter controlling the steepness of the curve (we will set $\gamma = 0.5$). Function *F* thus changes between 0 and 1. It can also be viewed as capturing relative differences in valuation of the collective goods by individuals of different rank^42,43^.

#### Strategy revision

After the collective action stage, each individual can revise her strategy with probability $\mu$, which measures the speed of the evolution. The approach we have chosen is to optimize the utility function via myopic optimization, i.e., pondering different possible actions while keeping all others’ decisions the same as in the previous round. In case an individual does revise her strategy, with probability $1-e$ an action *x* will be chosen such that $u_\eta$ is maximum. Otherwise, a random selection of *x* is selected with probability *e*.

#### Fitness

In this stage, the *i*th individual’s fitness, $w_i$, is defined by setting $w_i=1+\pi ^{*}/Q_{gen}-c_{opt}(1-\eta )-c_{int}\eta$. Here, $\pi ^*$ denotes the accumulated payoffs, $\pi ^*=\sum _j \pi _{CA, j} + \Delta _j$, the sum running over all rounds, with $\Delta _j$ being the the quantities earned or lost in each round *j*. The parameter $c_{opt}$ measures the cost of optimizing the payoffs instead of following the norm, and $c_{int}$ measures the cost of internalizing the norm.

#### Reproduction

Finally, reproduction is implemented proportionally to the individual’s fitness and the group’s collective effort. Each new group in the next generation will be descendant of one of the previous with probability proportional to the collective effort of the group, *P*, across *Q* rounds. Within each group, *n* parents will be chosen with probability proportional to their fitness, yielding *n* descendants. One individual can be chosen multiples times to yield descendants. Each descendant is subject to a random mutation on the attribute $\eta$. Offspring production is followed by random dispersal of half of the offspring, interpreted as females. The strategy revision, fitness, and reproduction steps, which are the ones introducing evolutionary dynamics in the individuals’ behavior, are taken from reference^48^.

In summary, our model combines the idea of fights feeding back into a hierarchical structure from Refs.^15,25^ with the participation in cooperative tasks and the possibility of social norm internalization from Refs.^42,48^, from where we have also taken the evolutionary dynamics for our simulations. We now turn to the discussion of the insights gained with this proposal on the arising of cooperative, structured societies.

## Results

In the following, we present the results of agent-based simulations of our model. We have considered two types of scores: one in which they can take any value, and another one in which they are restricted within an interval of integer values. Below we discuss the main features of our model, namely the final distributions for the scores, the payoffs and their relationship with the scores, the amount of cooperators, and the norm internalization process. Given the large number of parameters of the model, we vary those that are more relevant to our research question, keeping the rest unchanged. Specifically, the parameters we keep constant are the following: $T = 10{,}000$, $Q = 40$, $G = 500$, $e = 10\%$, $b = 1$, $c = 1$, $v = 1$ and $\mu = 75\%$, and $c_{opt}=c_{int}=0.1$. We choose similar parameters to those from Ref.^48^ to be able to compare the results of both models as needed.

### Unconstrained model

In the unconstrained model an individual’s score can increase and decrease without any restrictions, without upper and lower bounds for *r*. If we identify the score with somewhat similar to a physical force, this is a somewhat unrealistic situation, but it will allow us to gain some first insights on the outcome of our simulations.

**Figure 2:**
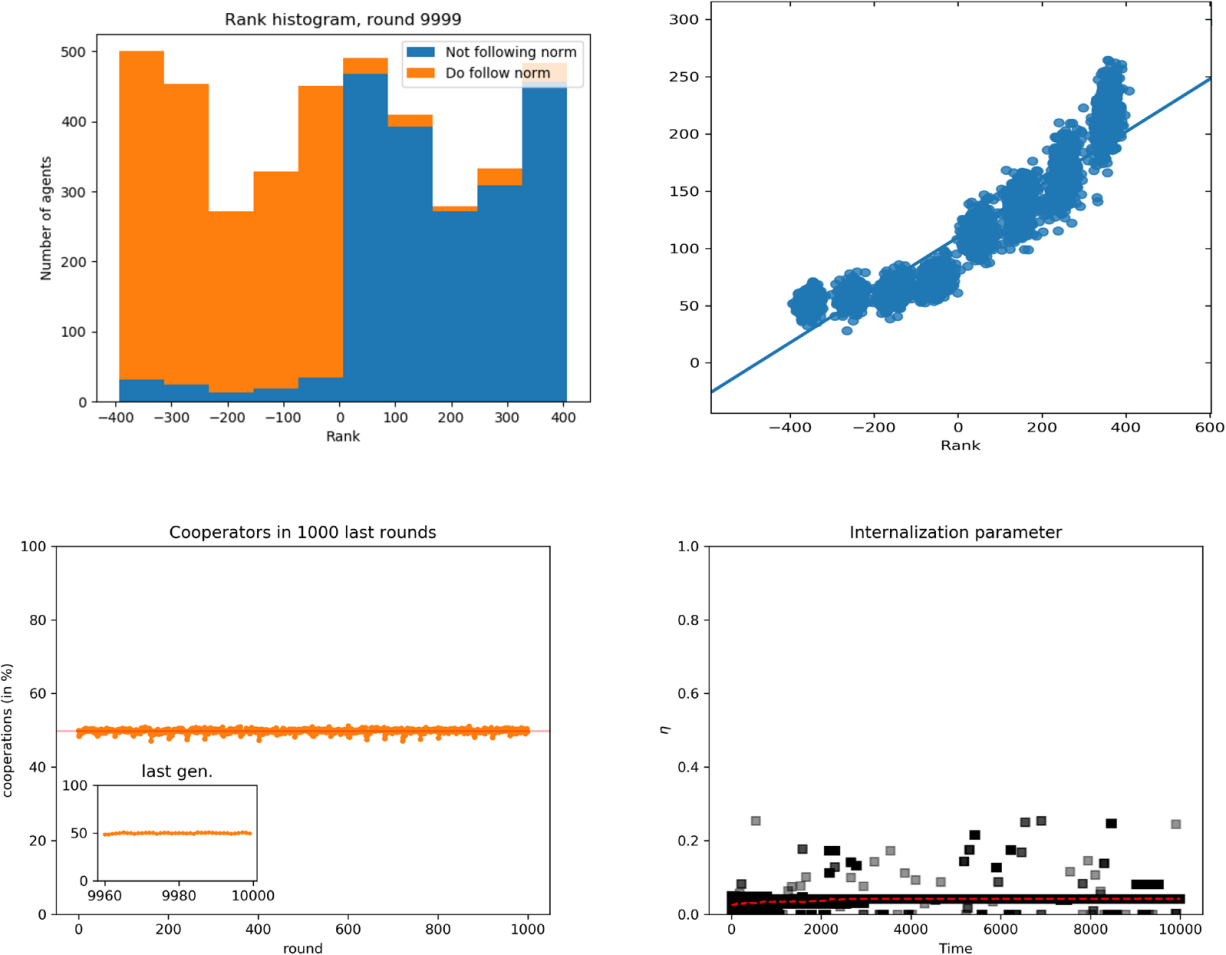
Simulations for unconstrained scores, small groups ($n=8$). Top left: histograms of scores aggregated over all groups. Blue indicates free riders (that do not follow the norm of cooperating), red indicates norm followers (cooperators). Top right: payoff as a function of the score. Line is the result of a linear regression. Bottom left: average fraction of cooperators as a function of time. Bottom right: average value of the norm internalization attribute as a function of time.

Figure 2 presents results for small groups $(n=8)$, aggregating over all groups. The score distribution is (almost) symmetrical with respect to $r=0$, whereas the higher the score, the higher the accumulated payoffs. For negative scores, the dependence of the payoff on the score is weaker, but for positive scores, payoffs increase rapidly with score, as can be seen from the plot. Thus, individuals are more or less evenly distributed among scores, but half of the population, those with negative scores, have significantly fewer payoffs than the rest: As can be seen, the highest-ranked individuals may have substantial payoff differences among them, whereas the lowest-ranked individuals have more homogeneous payoffs and similar to those with $r=0$. The reason for this is that individuals who won in the first rounds saw their scores increased, while those who lost were relegated to negative scores. These fundamental differences are subsequently amplified by the positive feedback loop *à la* Bonabeau^15^ ,leading to a ranking of agents with positions stable in time, i.e., there is no social mobility at all. Hence, subsequent evolution does not affect the score distribution, but does affect payoffs: Individuals with larger scores have been winning many fights, and as a consequence, they have accumulated more payoff.

Having seen that the fight mechanism does lead to a stable hierarchical structure in the society correlated with payoffs, we can now focus on our main question, namely the interplay of hierarchy, cooperativeness, and social norm internalization. In Fig. 2 we can observe that the cooperation value per round is around $50\%$, with the other half of the population defecting in the collective action. This can be interpreted as half of the population being oppressed by the other half: exploited individuals (those with negative scores) cooperate in the collective action and those exploiting them (having positive scores) reap their payoffs. Indeed, low ranked individuals, with a very slim chance to defeat higher ranked ones, must cooperate to get some payoff, which they keep when they are not picked for a fight and defeated. This agrees with the results in the score histogram, where we observe that the top half part of the population is the one not cooperating (not following the norm, choosing $x=0$) and the bottom half is the one contributing to the collective action (following the norm, $x=1$). In addition, there is very little internalization of the norm, mostly because those who might be better off by internalizing the norm in their utility function have very low fitness and practically never reproduce. Of course, a few lower-ranked individuals do show norm internalization, with values around $\eta =0.3$, but these are clearly not the general case.

The aggregate results we have just discussed describe well the evolution of individual groups. Section S1 in the SI shows examples of simulation results for individual groups. In brief, what we observe is that the division in high-ranked agents that do not follow the norm and low-ranked agents that do takes place in most groups, albeit subject to a degree of noise as sometimes, for instance, there is an agent that is high-ranked and follows the norm (cf. Fig. S1b). In any event, the fraction of mismatched agents is always low. Similarly, the correlation between score and payoff applies at each individual group, with a small degree of noise. At the same time, agents with more probability to reproduce and pass offspring to the next generation are those with higher scores (cf. Figs. S5 and S6). We can thus be confident that the aggregate results are a good picture of what happens in individual groups, which are the units whose social structure is relevant.

For larger groups, namely $n=24$, there are two main differences with the results reported so far: First, the score distribution changes and, second, the average cooperation level follows a different behavior. Regarding the aggregate score distribution, for groups with 24 individuals, we cannot say that the scores are evenly distributed as before. The distribution is more similar to a Gaussian, with many agents in the middle zone and only a few of them with extreme (positive or negative values). This is probably due to the length of the lifetime of the group, which is the time scores have to evolve. Having more individuals means that more fights would be needed to organize them in a more linear form. As for the cooperation level, at every generation it increases from the initial random level at $50\%$ up to $\sim 90\%$, and then decreases to the initial value, around $50\%$, jumping again at the next restart of the population as the ranking between agents sets in. There are no changes regarding the internalization level of the norm, which is similar to the previous case and for similar reasons, as well as with respect to the relationship between the payoffs and the scores. Therefore, our main conclusion is that also in larger groups there is no promotion of cooperation and the norm does not become internalized. On the other hand, as with small groups, results for individual groups are very similar to those reported for the aggregate, cf. Figs. S2, S4 and S6 in the SI.

**Figure 3:**
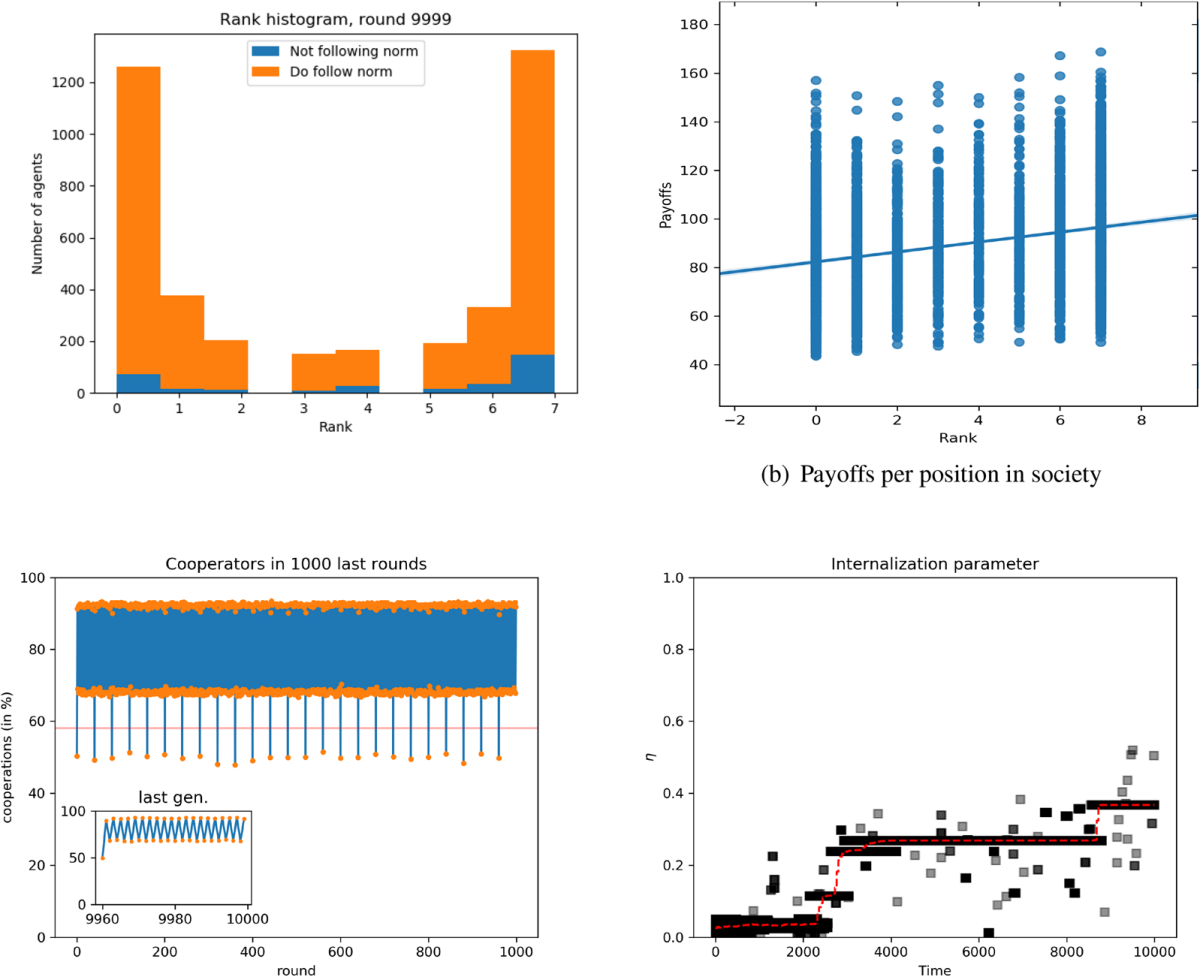
Simulations for exogenously constrained scores, small groups ($n=8$). Top left: histograms of scores aggregated over all groups. Blue indicates free riders (that do not follow the norm of cooperating), red indicates norm followers (cooperators). Top right: payoff as a function of the score. Line is the result of a linear regression. Bottom left: average fraction of cooperators as a function of time. Bottom right: average value of the norm internalization attribute as a function of time.

### Exogenously constrained model

In this section, we consider a slightly more realistic version of our model, in which scores are restricted to the interval [0, *n*], *n* being the group size. This constraint is imposed externally, hence the name ‘exogenously constrained’; we will consider below another variant of the model in which scores decay with time resulting in an endogenous constraint of their values. As we will see, constraining scores gives rise to significant changes with respect to the unconstrained version.

Figure 3 shows the score distribution, in which the majority of individuals end up in the maximum and the minimum, with few agents occupying intermediate scores. This occurs because as the system evolves, fights won (respectively, lost) feedback on score, leading to higher (respectively, lower) scores, pushing individuals to the maximum (respectively, minimum) possible values. On the other hand, having many individuals in the lowest and highest rankings leads to a much lower impact of score on payoffs, as shown in Fig. 3, where no significant relationship between score position and payoff is observed. The reason for this is that at both extremes of the hierarchy there are many individuals with the same score, and fights between them lead to random results. This is in stark contrast with the unconstrained case, where the large differences in score decided practically every fight and induced a correlation with payoffs.

As can be seen from Fig. 3, cooperation level starts at around $50\%$, the initial random assignment of cooperation-defection in each new generation, and rapidly increases a high value. Subsequently, it oscillates between 70 and 90%. It is important to note that the inset of the plot corresponds to the last 1,000 rounds of evolution, where the mean value of the norm has reached a value around 0.4. as also shown in Fig. 3. Indeed, contrary to the unconstrained model, we find that the norm is internalized to a high value, thus increasing the cooperation level to high values. At the level of individual groups, we observe that the small fraction of individuals who do not follow the norm can be anywhere in the hierarchy, most likely because their small number makes them not very relevant in terms of the dynamics of the society.

For the exogenously constrained model, when we go down to the level of individual groups, there is a larger variability in behaviors, still within the same overall picture. There may be low-ranked agents who do not cooperate or, on the contrary, cooperative agents with the highest rank, cf. Figs. S7 (a) and (b) in the SI. The most important point to notice with respect to the individual groups of size $n=8$ is that agents have a fitness distribution that is not correlated with their position in the hierarchy (cf. Fig. S11). This arises because the limits to the scores allow for larger mobility of the agents within the scores: high-ranked agents at one time may become low-ranked after a few, unlucky rounds. We have confirmed that this is indeed the case by checking the evolution in time of the scores (cf. representative examples in Figs. S13a). In turn, the fact that agents spend time in low scores leads to an increase of the norm internalization attribute, as we have observed in the aggregate. We believe that this mobility is the reason for the increased cooperation and norm internalization found in the aggregate (cf. Fig. 3). Indeed, as agents spend part of the lifetime of their group in lower scores, they generally cooperate and their degree of norm internalization increases. Subsequently, the small differences in payoff between higher-ranked and lower-ranked agents makes them reproduce with similar frequencies, allowing for the norm to continue internalizing in the next generation.

**Figure 4:**
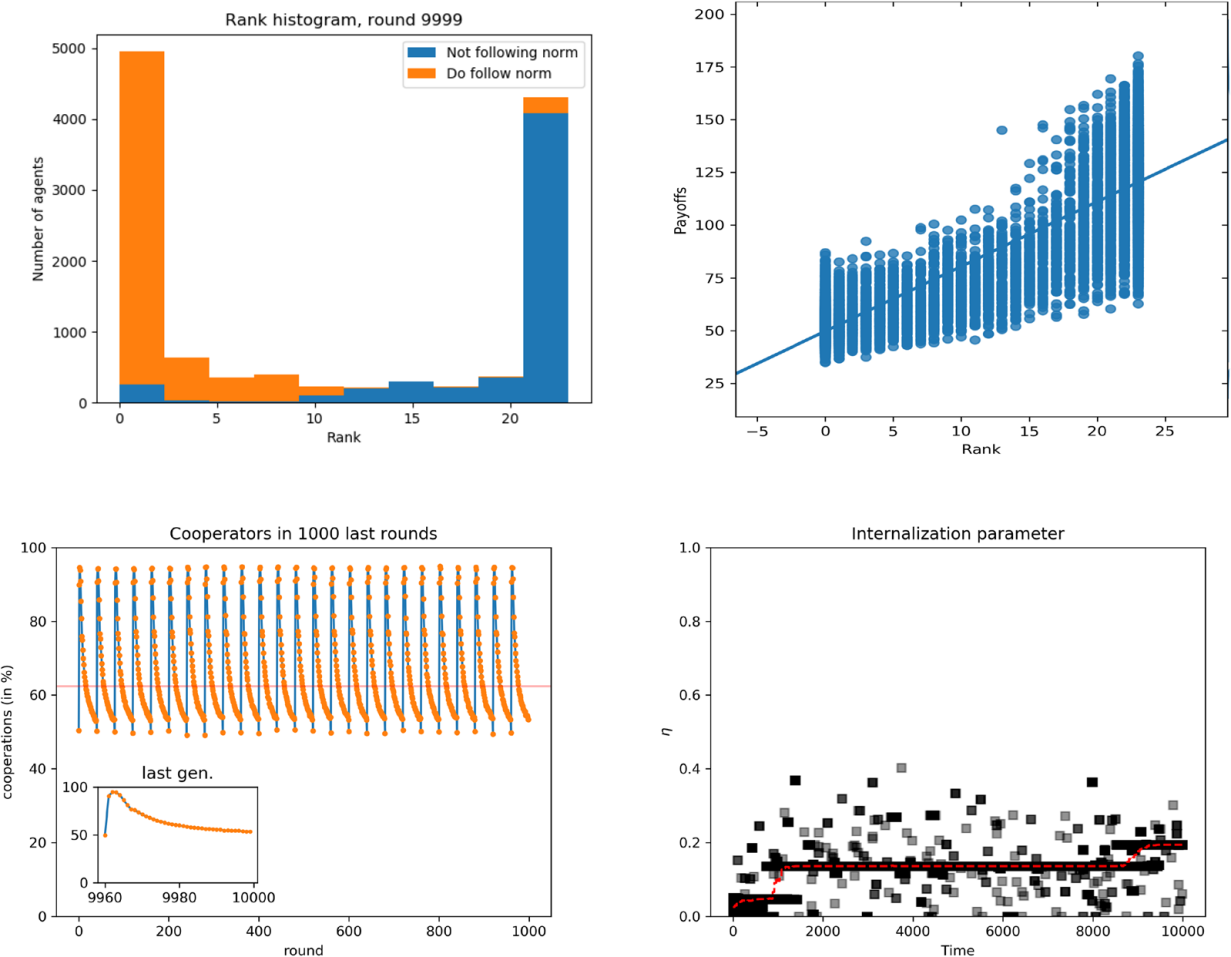
Simulations for exogenously constrained scores, large groups ($n=24$). Top left: histograms of scores aggregated over all groups. Blue indicates free riders (that do not follow the norm of cooperating), red indicates norm followers (cooperators). Top right: payoff as a function of the score. Line is the result of a linear regression. Bottom left: average fraction of cooperators as a function of time. Bottom right: average value of the norm internalization attribute as a function of time.

As with the unconstrained model, the outcome of the simulations changes again when the group size increases. This is shown in Fig. 4, where it can be noticed that the score distribution is similar to the previous case, with an even more bimodal character, but now the payoff distribution is more clearly dependent on the score, most likely due to the fact that there are more individuals and the range of possible scores is larger. The fact that now the payoffs depend more on the score leads to a situation with less cooperation and less norm internalization: Indeed, cooperation tends to the same values as in the unconstrained model: the dynamics follows the same pattern as in the case of small groups, but now the decay in each generation reaches values close to 50%. We also observe that the norm internalization achieves lower values, though still more significant than in the constrained model. This change has another consequence, namely that we go back to a situation in which high ranked individuals do not follow the norm while low ranked ones do as in the case of the unconstrained model. For the case of large, individual groups, the phenomenon of mobility within scores is quite less marked, mostly because now agents can have a larger range of scores, in agreement with the fact that there is less norm internalization and higher dependence of the payoff on the scores.

### Endogenously constrained model

For the sake of making our model more realistic, we now consider another version in which scores decrease following a certain rule. While this decay does not impose any hard bound to the score values, it effectively leads to a constrained range for them, hence the name ‘endogenously constrained’ model. This version consists of the same stages as before (collective action, conflicts, and reproduction) plus an additional one, the time evolution of the scores, which obeys the following dynamics: $r_{t+1}=(1-\lambda )r_t + \Delta r$, with $0 \le \lambda \le 1$, rounding $r_{t+1}$ to the closest integer value. This rule implies that, in the absence of any interaction, scores decrease to a fraction of their initial value $((1-\lambda )r_t)$, whereas the results of the fights affect them through the term $(\Delta r)$. Consistently with the previous versions of our model, we set $\Delta r=\pm 1$, depending on whether an individual wins, $+1$, or loses, $-1$, a fight. This update rule can be interpreted as follows: when the value of $\lambda$ is low, the next competitive score will be close to the previous one, and the benefits of scaling in the ranking by increasing one’s score through fighting last longer. When the value of $\lambda$ is high, every new score will be closer to $r=0$, making the society more egalitarian every round and strongly limiting the score benefits in time. Thus, we have a new parameter $\lambda$, whose effect we consider below.

**Figure 5:**
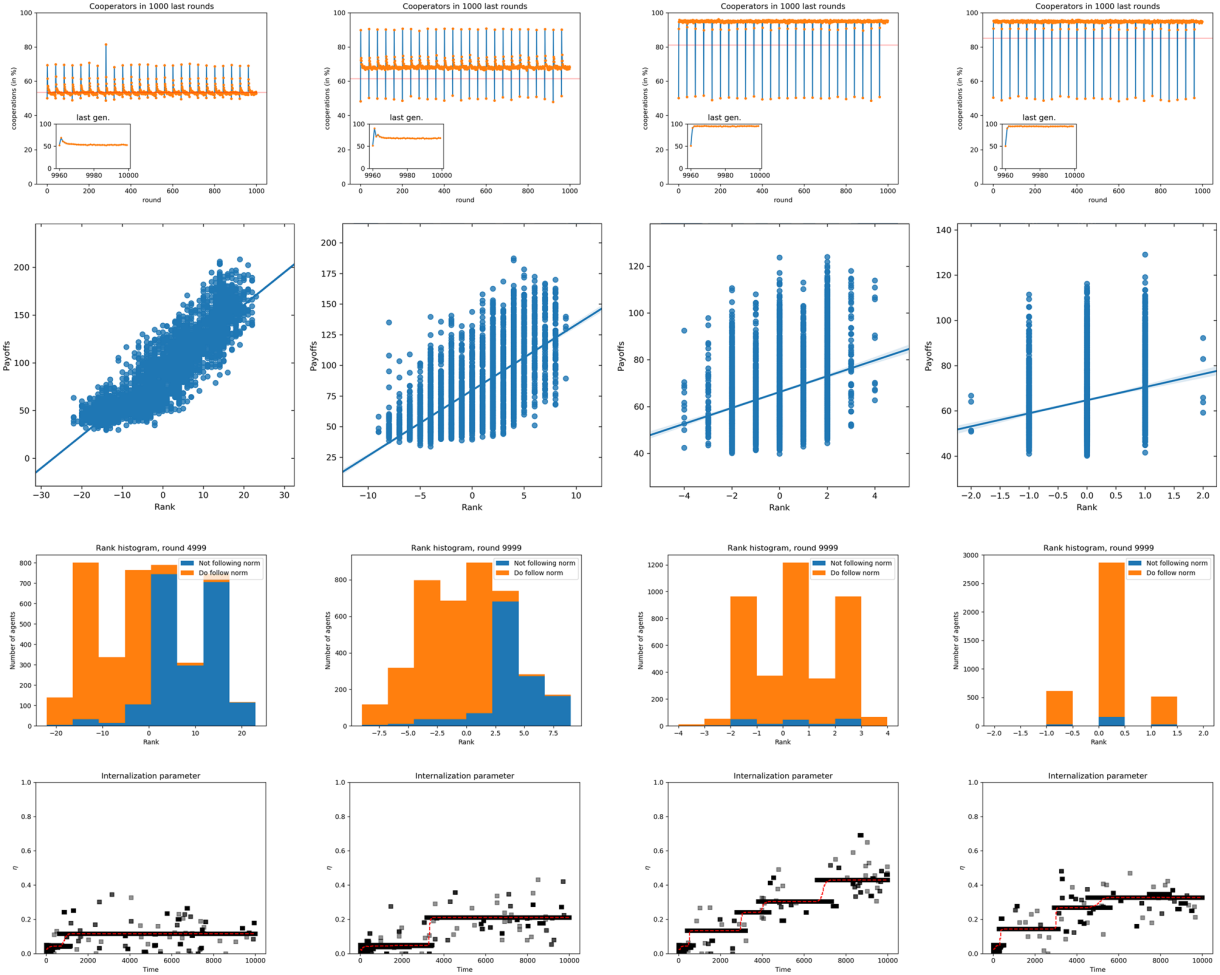
Top to bottom: Histograms of scores aggregated over all groups; blue indicates free riders (that do not follow the norm of cooperating), red indicates norm followers (cooperators). Payoff as a function of the score; line is the result of a linear regression. Final distributions of scores for different values of $\lambda$. Internalization attribute evolution for different values of $\lambda$. Left to right, $\lambda =0.1, 0.25, 0.5$ and 0.75.

Figure 5 collects the results for the endogenously constrained model in small groups, showing that, as we have just discussed, the higher $\lambda$ the more egalitarian the society, to the point that for $\lambda =0.75$ only three different scores exist. The structure of the society is different from that of the exogenously constrained model, in the sense that now we observe that most individuals concentrate in intermediate scores and only a few of them have extreme (positive or negative) values. At the same time, cooperation grows with $\lambda$, reaching almost full cooperation for $\lambda =0.5$ and higher. The differences in payoff are large for small $\lambda$, whereas upon increasing $\lambda$ the influence of the score on the payoffs becomes very limited. As regards norm internalization, it increases with $\lambda$, as can be seen in Fig. 5: internalization is higher for larger values of $\lambda$, reaching values similar to those of the exogenously constrained model. We believe that these results arise from the fact for such limited range of scores they are no longer decisive to decide the outcome of the fights. In this model, most agents have zero score, particularly for large $\lambda$, and therefore fights are not so relevant anymore, as most participants randomly win half of them. This is in contrast with the exogenously constrained model, where agents are concentrated in the minimum and maximum values, and they win every fight with the lower half of the population.

**Figure 6:**
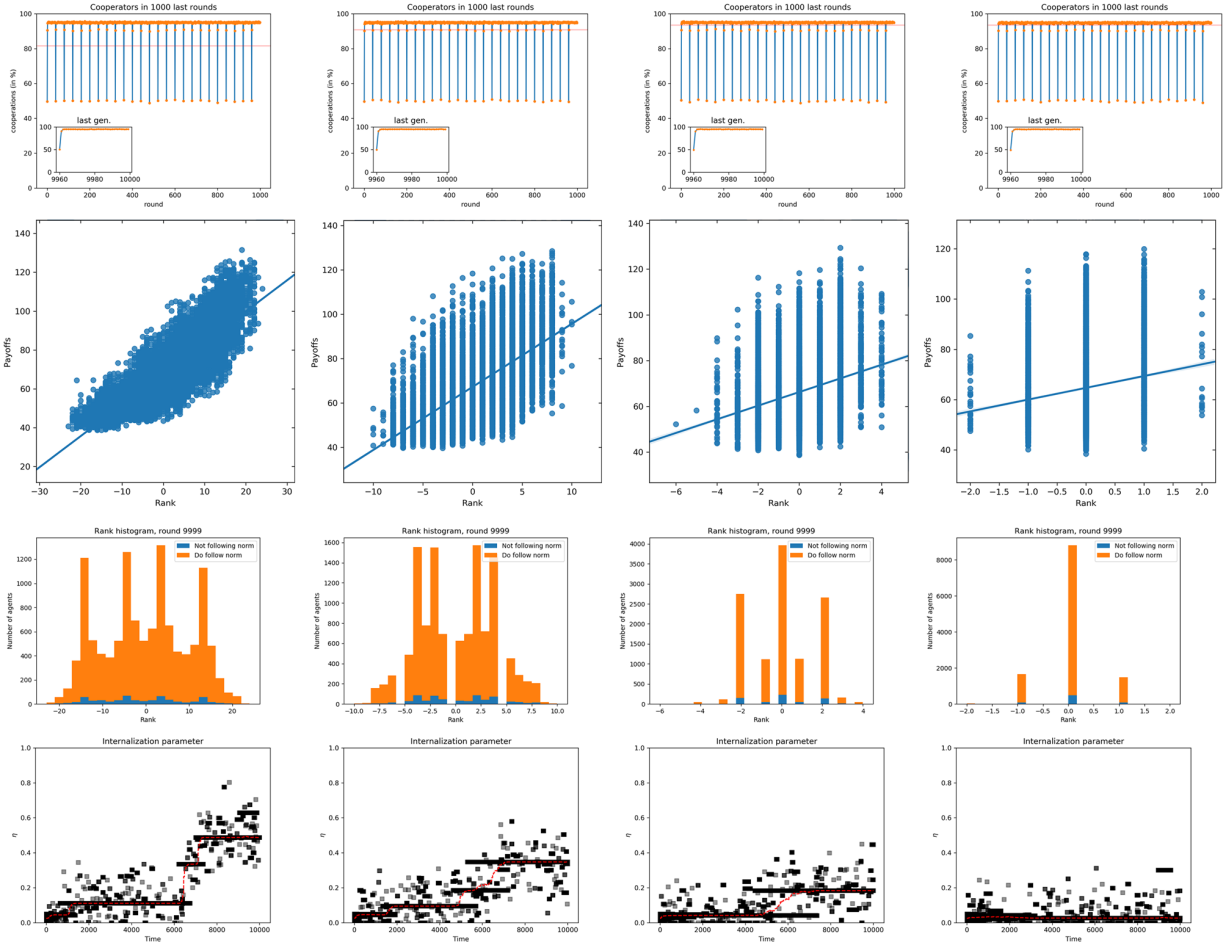
Simulation results for large groups, $n=24$. Top to bottom: Histograms of scores aggregated over all groups; blue indicates free riders (that do not follow the norm of cooperating), red indicates norm followers (cooperators). Payoff as a function of the score; line is the result of a linear regression. Final distributions of scores for different values of $\lambda$. Internalization attribute evolution for different values of $\lambda$. Left to right, $\lambda =0.1, 0.25, 0.5$ and 0.75.

The changes in the results when group size increases up to $n=24$ can be seen in Fig. 6. While the distribution of scores is very similar to the case of small groups, the dependence of payoffs on scores is essentially the same as before. As for the behavior concerning norm internalization, it is the opposite to the one observed in Fig. 5: when $\lambda$ increases, the attribute $\eta$ is less internalized. At the same time, the cooperation level is very high for all values of $\lambda$. We believe that for large groups, the large $\lambda$ results are comparable to those in Ref.^48^. Given that most individuals are equal, fights become statistically irrelevant, and therefore the situation is similar to that model, with no fights and no punishment. Thus, in agreement with this previous work, we see very little norm internalization for large groups with endogenously constrained scores. The results from the individual groups (cf. Figs. S15 and S16 in the SI) agree in general with this picture; the fact that the mobility between ranks is somewhat lower for larger groups (cf. Figs. S17 and S18) may also be another reason for the smaller norm internalization in that case.

## Discussion

In this paper, we have introduced an agent-based model showing how a hierarchical structure may emerge in a population of initially egalitarian individuals, and how this structure affects within-group cooperation. As we have seen, the emergence of hierarchies is a robust feature of the model observed in both versions we considered in our research: unconstrained or constrained scores, and both for small or large groups. The specific score structure and other features of the model do depend on the version of interest. Thus, when score values are unconstrained, leading to substantial, time-independent probabilities of winning (or losing) fights, payoffs and ranking are very correlated: the higher the score, the larger the payoffs, for small and large groups, which is a characteristic of hierarchical structures. Cooperation is limited to lowest score individuals, and about half of the individuals cooperate. This takes place without any internalization of the norm whatsoever and arises because of purely material interest, i.e., agents try to maximize their payoff by generating resources even if they might be robbed via fights.

On the other hand, when we impose an external constraint on the scores, avoiding indefinite growth of fight capability, society becomes structured, polarized, in fact, with most individuals being either at the top or the bottom of the ranking. This structure turns out to be compatible with high cooperation levels. However, contrary to the unconstrained model, in this case, cooperation takes place via norm internalization. It is also observed that the degree of norm internalization is smaller for larger groups, and in this case, it is internalized mostly by lowest-ranked individuals. In this society, individuals experience rapid transitions between scores: when they go down in the hierarchy, they cooperate because the individuals who are higher than them in the hierarchy can take the payoffs from them, and they cannot reciprocate. In turn, this leads to norm internalization when individuals experience a phase of having low scores. Interestingly, an interpretation of these results is that this dynamical hierarchy can play the role of the punishment phase in Ref.^48^.

We have also introduced the possibility that constraints arise endogenously, decaying when individuals do not fight. In this case, norm internalization takes place also for small groups, and it is not an artifact of the arbitrarily imposed limits. The structure of the society is different in this case, with a large fraction of the population living in the region of intermediate scores. In the case of larger groups, this makes fights practically irrelevant, as many individuals win or lose them 50% of the times, making the model comparable to that studied in Ref.^48^, where the norm did not internalize for large groups.

Generally speaking, all three versions of the model produce hierarchical structures in small groups, but only when scores are constrained, be it exogenously or endogenously, there is a sizable degree of norm internalization. As was the case in Ref.^48^ (in the so called us-vs-them situation, competition between groups) larger groups pose more difficulties to norm internalization, although in the case of the exogenously constrained model this occurs to a lesser extent. Therefore, the main conclusion we can draw from our models is that it is possible to observe a transition from an egalitarian society—with all individuals ranked equally—to another one where there is a hierarchy, but, at the same time, there are high levels of cooperation. We want to stress that this does not occur only for a very restricted set of parameters and attributes, but coexistence of cooperation and hierarchy arises in a wide range of values. Thus, we varied the group sizes ($n=8, 16, 24$), the multiplier of the collective effort ($b=2, 4$), the value of cooperation of the utility function ($\nu =1, 1.5$), the probability that the winner of the fights is the one with the higher rank (0.9, 0.75, 0.5), including changing the dependence of the probability on the rank, the probability to revise the strategy ($\mu =0.9, 0.75, 0.5$), and the error rate of optimization ($e=0.1, 0.25, 0.5$). Finally, we also studied different choices for the decay parameter in the endogenously constrained model ($\lambda =0.1, 0.25, 0.4, 0.5, 0.75, 0.8$), and the cost of optimizing the payoffs and the cost of internalizing the norm ($c_{opt}=c_{int}=0.1, 0.25$). The number of parameters makes it very cumbersome to carry out a full sensitivity analysis, but we believe that these checks allows us to be confident that the outcome of the model is very general and not strongly dependent on the parameters.

Our finding that hierarchy can arise in a cooperative society is in contrast with the experimental observation that hierarchy might be detrimental for cooperation in Ref.^40^, but consistent with the fact that cooperation may be compatible with rankings arising from cooperative action^41^. Interestingly, the main difference between the nature of fights in our model and in Ref. ^40^ is the lack of feedback from fights to hierarchy in the latter: experiments were carried with a fixed score assigned to each individual. This strongly suggests that a key feature behind the coexistence of hierarchy and cooperation may be the dynamic character of the score. Besides, at least for small groups with a realistic (constrained) ranking system, this may require an additional ingredient, namely the possibility of internalizing a norm that favors cooperativeness. In this respect, our model is aligned with recent work^51^ showing that societies developed in regions where agriculture—i.e., solving the collective action problem—was practiced for longer, providing more time for norms to emerge, and conflicts were more intense creating a stronger selection pressure. Further research is needed to assess appropriately the mechanisms allowing cooperation in strongly hierarchical societies, and we hope that this paper stimulates such research.

## Supplementary information

Supplementary Information.
