# Supplementary material for: Cooperation, social norm internalization, and hierarchical societies
Source: Sci Rep. 2020 Sep 21;10:15359. doi: 10.1038/s41598-020-71664-w (PMC7506014; doi:10.1038/s41598-020-71664-w)
Supplement: Supplementary file 1 — Supplementary Information. [file 41598_2020_71664_MOESM1_ESM.pdf]

Supplementary Information: Cooperation, social norm internalization, and hierarchical societies

Pablo Lozano[1,2,*], Sergey Gavrilets[3], and Angel Sánchez[1,2,4,5]

[1]Grupo Interdisciplinar de Sistemas Complejos, Departamento de Matemáticas, Universidad Carlos III de Madrid, 28911 Leganés, Madrid, Spain
[2]Unidad Mixta Interdisciplinar de Comportamiento y Complejidad Social (UMICCS), Spain
[3]Department of Ecology and Evolutionary Biology, Department of Mathematics, National Institute for Mathematical and Biological Synthesis, Center for the Dynamics of Social Complexity, University of Tennessee, Knoxville, TN 37996, USA
[4]Institute for Biocomputation and Physics of Complex Systems (BIFI), University of Zaragoza, 50018 Zaragoza, Spain
[5]UC3M-Santander Big Data Institute (IBiDat), Universidad Carlos III de Madrid, 28903 Getafe, Madrid, Spain
[*]Corresponding author: pablo.lozano@uc3m.es

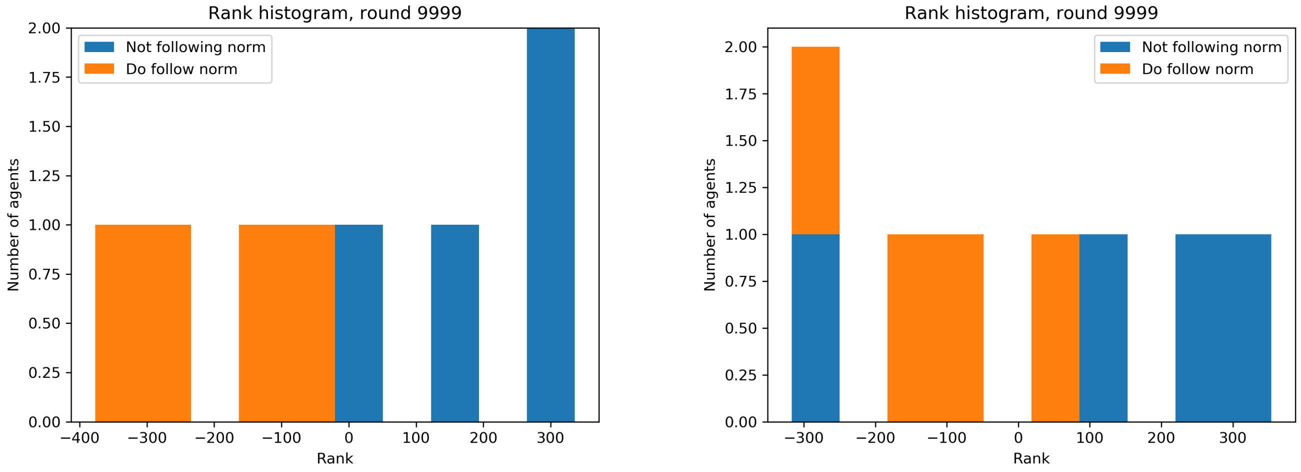

(a) Rank distribution—group 94 (b) Rank distribution—group 326

Figure S1: Rank distributions for the unconstrained model—individual, small groups.

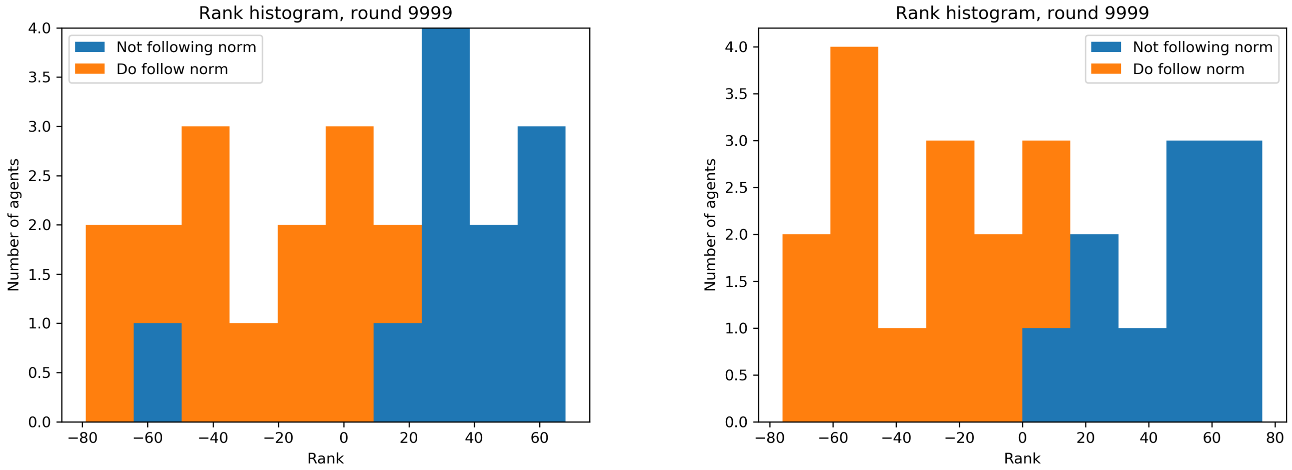

(a) Rank distribution—group 39 (b) Rank distribution—group 213

Figure S2: Rank distributions for the unconstrained model — individual, big groups.

S1 Unconstrained model: Individual groups

The results reported in the main article offer an overall picture of the whole population, by aggregating the results of different groups. However, it is essential to take the study one level down and look at processes and mechanisms at the individual group level, to assess to what extent the aggregate results describe them well. Therefore, we now look at this individual group level, beginning with Figure S1 (a), where we can see representative examples showing that, as previously mentioned, most of the individuals in the upper half do not follow the norm of cooperating in the collective action. In contrast, the rest of the individuals follow the norm. This is not always true, inasmuch of in Figure S1 (b) there is an individual with rank $\gg 0$ who is following the norm, and an individual with rank $\ll 0$ who is not following the norm. Therefore, the behavior is subject to noise, but the proportion of individuals *misbehaving* for their rank is very low if we compare these figures with Figure 3 (c) from the main article, where the aggregated behavior is shown. The same applies to the case of bigger groups but, due to the increased number of individuals, the behavior is more consistent, and those who have a high (respectively, low) rank and cooperate (respectively, do not cooperate) are less noticeable.

Also, within this perspective of what goes on in each group, we observe that the payoffs' distribution correlates with the expected behavior: within the groups, the individuals with higher rank have more payoffs

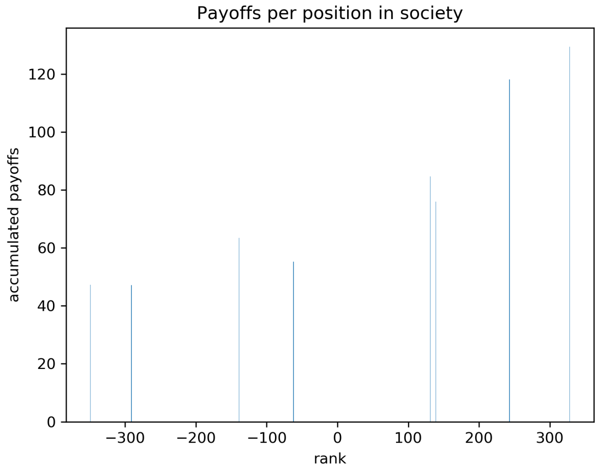

(a) Payoff distribution — group 94

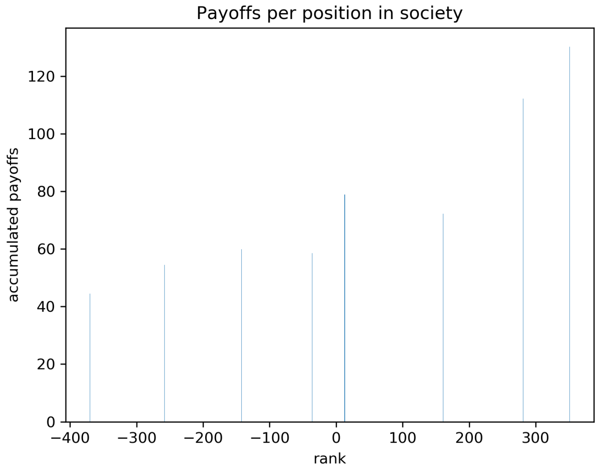

(b) Payoff distribution — group 240

Figure S3: Payoff distributions for the unconstrained model — individual, small groups.

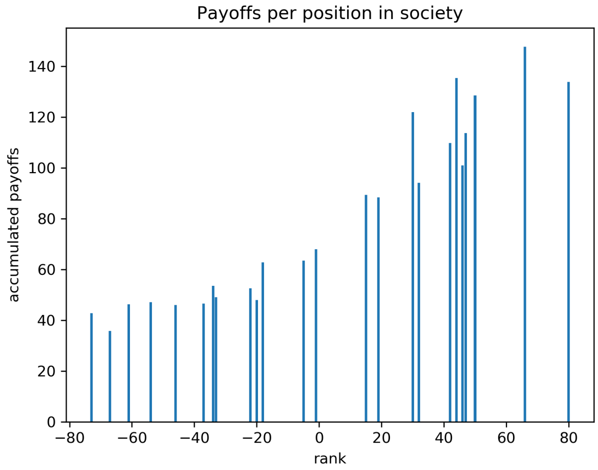

(a) Payoff distribution — group 63

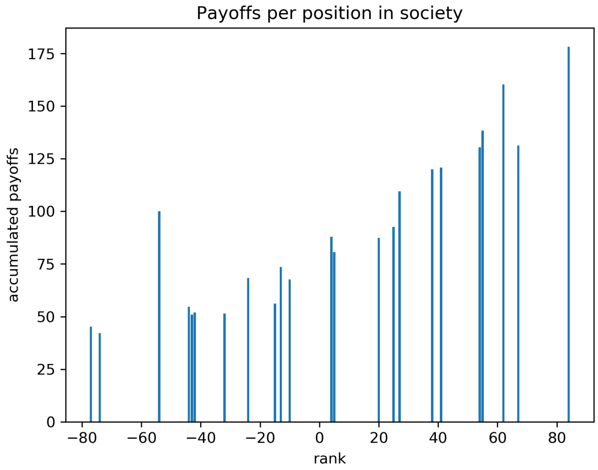

(b) Payoff distribution — group 93

Figure S4: Payoff distributions for the unconstrained model — individual, big groups.

than those in lower ranks (according to Figures S3 and S4). This is consistent with the fact that the higher the rank, the more probable it is for an individual to win a fight and, therefore, to accumulate more payoffs. There is still noise in the distribution, as in Figure S4 (b), an individual with low rank has more payoffs than those above it.

Finally, attending to the individual distribution of fitness values concerning the position in the hierarchy, Figures S5 and S6 show that the individuals with more probability of having offspring the next generation are those with the higher ranks. This happens in both small and big groups. With these figures and the ones in S1-S2, the individual behavior in the reproduction stage can be predicted: those who reproduce with higher probability are those who do not follow the norm, in agreement with the observed behavior of the internalization parameter in Figure 3 (b) from the main article. In any event, looking at these individual examples, and at the rest of those in the simulations, we can conclude that the aggregate behavior represents well the general features of individual groups.

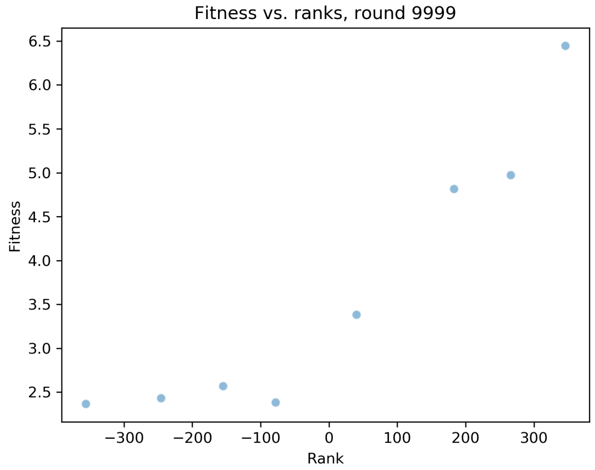

(a) Fitness vs ranks — group 47

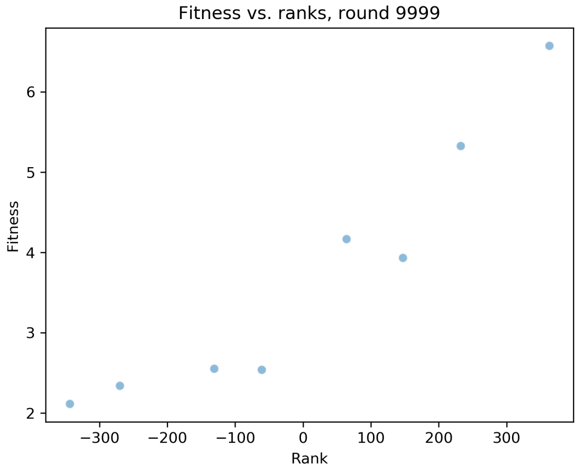

(b) Fitness vs ranks — group 141

Figure S5: Fitness vs ranks for the unconstrained model — individual, small groups.

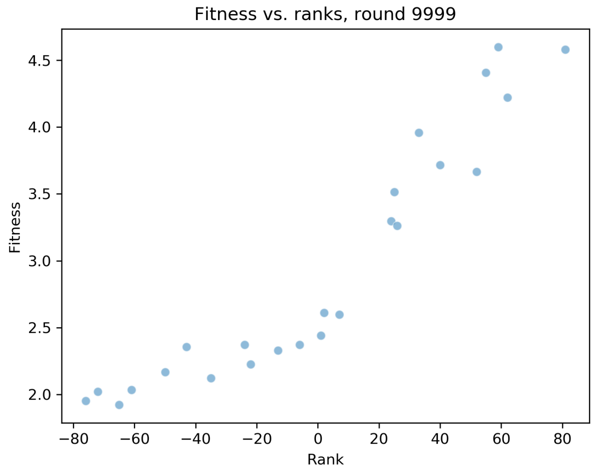

(a) Fitness vs ranks — group 55

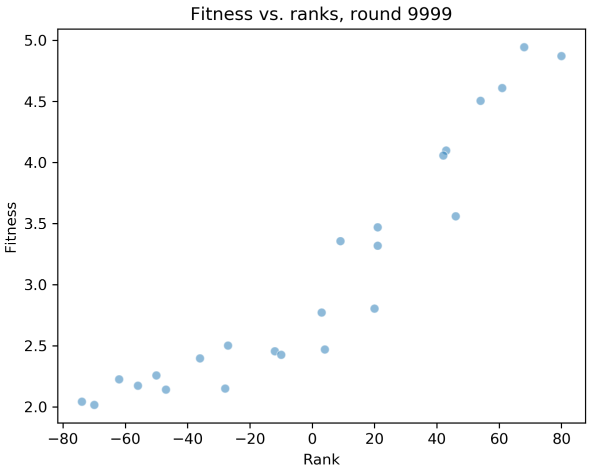

(b) Fitness vs ranks — group 154

Figure S6: Fitness vs ranks for the unconstrained model — individual, big groups.

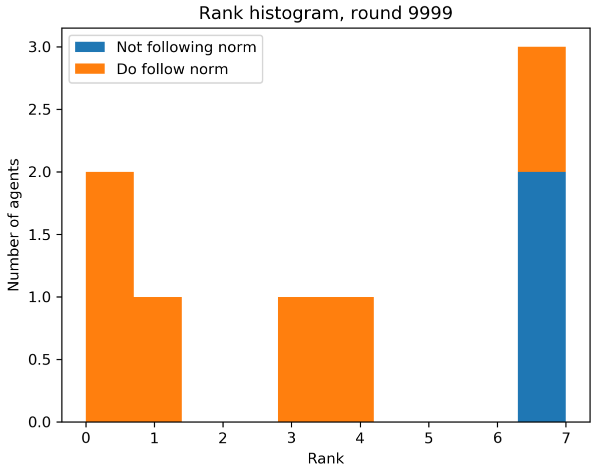

(a) Rank distribution — group 213

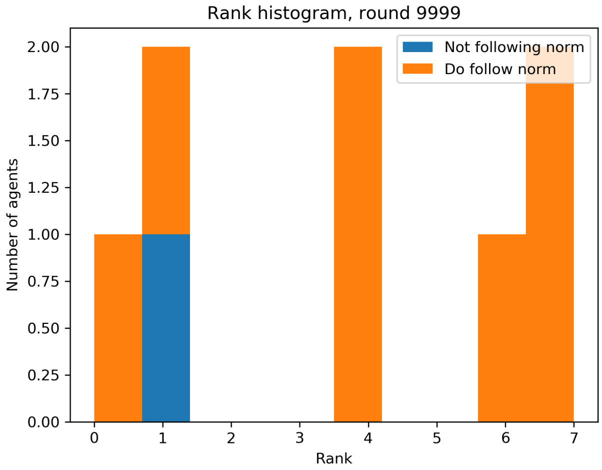

(b) Rank distribution — group 288

Figure S7: Rank distributions for the constrained model — individual, small groups.

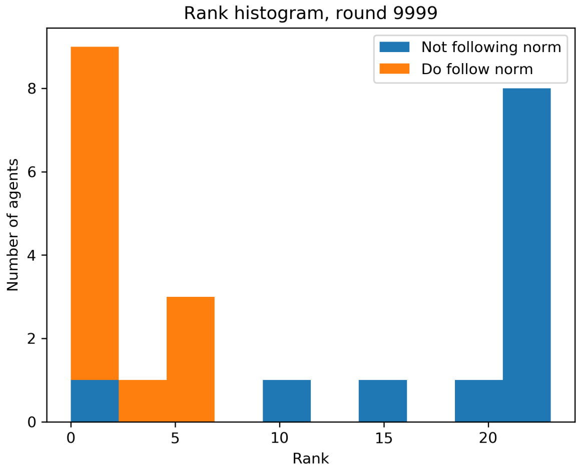

(a) Rank distribution — group 213

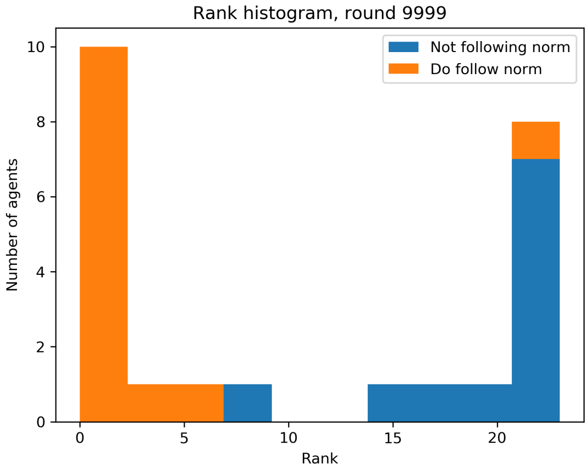

(b) Rank distribution — group 405

Figure S8: Rank distributions for the constrained model — individual, big groups.

S2 Exogenously constrained model: Individual groups

Figures below show the ranks distributions for individual groups within the constrained model with size $n = 8$ (Figure S7), and $n = 24$ (Figure S8). Groups have been randomly selected, and the behavior is consistent between all of them. As in figure S1, if the individual is in the lower part of the ranking, it is more likely to cooperate. However, there is a larger degree of variability in behaviors, as shown in the rest of the examples in this figure. There may be individuals with low-ranking who do not cooperate or, on the contrary, individuals that do cooperate even when they have the highest rank, as in figures S7 (a) and (b).

The difference between the constrained model and the unconstrained model can be seen in the payoff distributions from Figures S9 and S10. For the constrained model, all the individuals have, approximately, the same amount of payoffs when the groups are small (Figures S9), with an increment in the difference when the size of the group increases (see figures S10).

There exists a critical difference between this model and the previous, unconstrained one, that can be seen in Figures S11 and S12. The individuals in small groups (S11) have a fitness distribution that is not correlated with their position in the hierarchy. Some individuals have the highest fitness even when its rank is the lowest (see Figure S11 (a) or (b)). This translates into a higher probability of selecting an individual

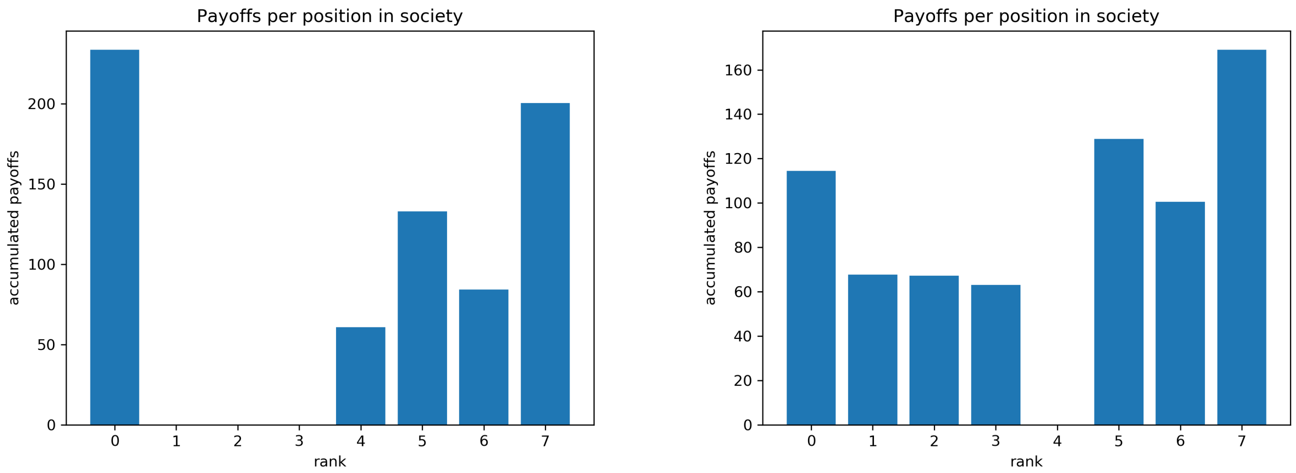

(a) Payoff distribution — group 260

(b) Payoff distribution — group 402

Figure S9: Payoffs distributions for the constrained model — individual, small groups.

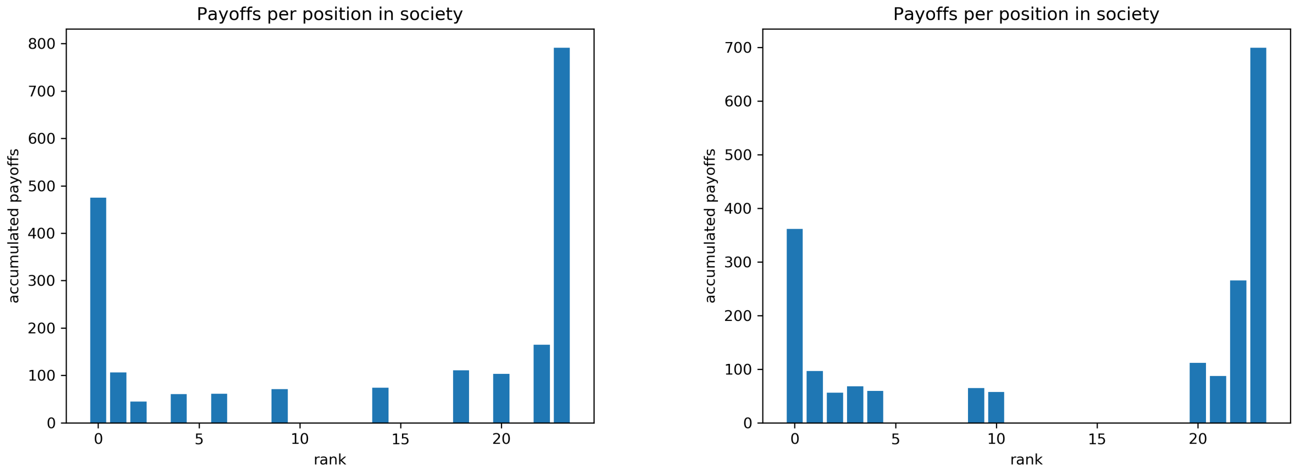

(a) Payoff distribution — group 340

(b) Payoff distribution — group 489

Figure S10: Payoffs distributions for the constrained model — individual, big groups.

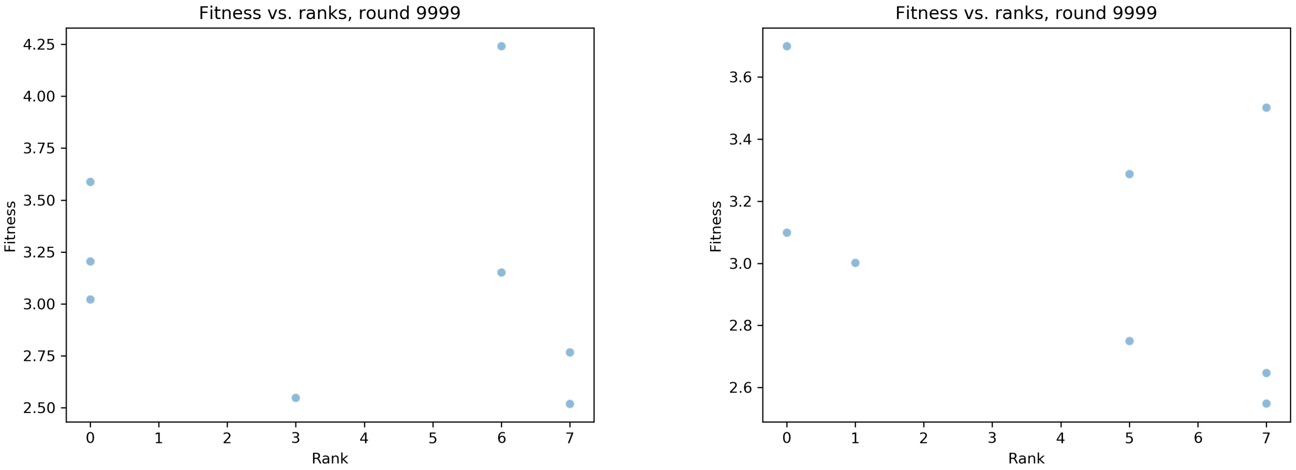

(a) Fitness vs ranks — group 67 (b) Fitness vs ranks — group 211

Figure S11: Fitness vs rank for the constrained model — individual, small groups.

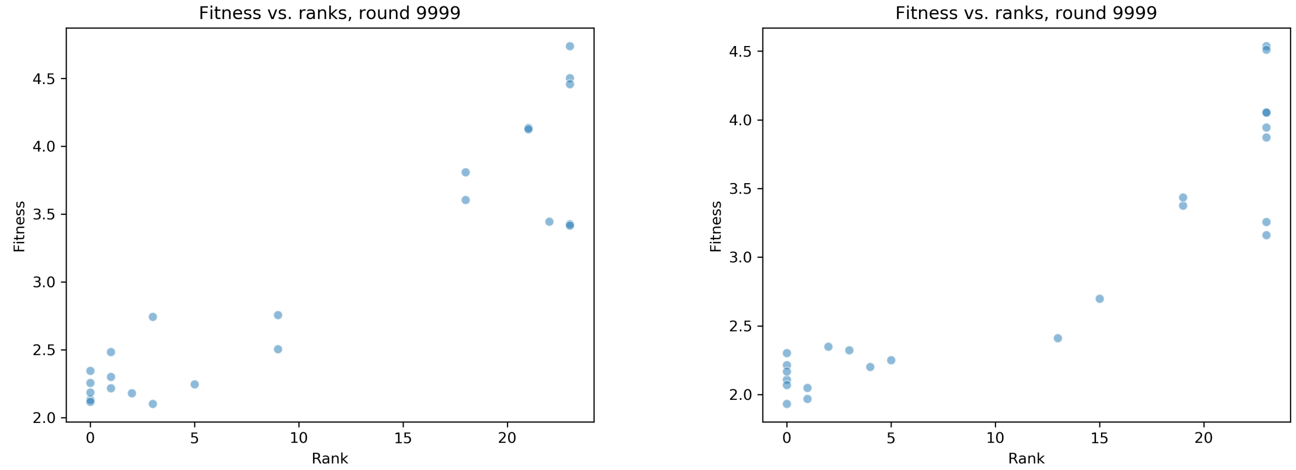

(a) Fitness vs ranks — group 382 (b) Fitness vs ranks — group 445

Figure S12: Fitness vs rank for the constrained model — individual, big groups.

with a low rank for reproducing (and thus, more probable that cooperates in the CA). The mechanism making this happen is the same that increases the internalization parameter in Figure 5 from the main article: the mobility of the individual within the hierarchy, from high ranks—where it can increase its accumulated payoffs, making it more probable to be selected at the individual level when reproducing—to low ranks—where it cooperates more due to its rank, and that makes its group increase its success in the CA problem, thus being more probable to be selected at the group level—. This does not happen when the group size is larger, as in Figures S12, so the increase in the size mitigates this effect.

We have looked at different trajectories from randomly selected individuals (Figure S13 and S14) to provide grounds for our hypothesis: moving individuals in the hierarchy increases internalization by forcing them to cooperate in low ranks. In these plots, two individuals from the same group are selected at random, and their trajectories are plotted. In Figure S13 (a) we observe different behavior from the ones in figures (b) to (b). In figure (a), the individuals exchange their positions: one of them increased its rank up to the maximum and then suddenly decreased it to the minimum possible. However, the second individual maintained a low rank while the first was on the highest possible rank; and slowly increased it up to the maximum in a few rounds. Individuals transitioning from low ranks to high ones and vice versa are indeed observed in these models.

On the contrary, in Figure S14 (a), we observe individuals who had a constant behavior of increasing or

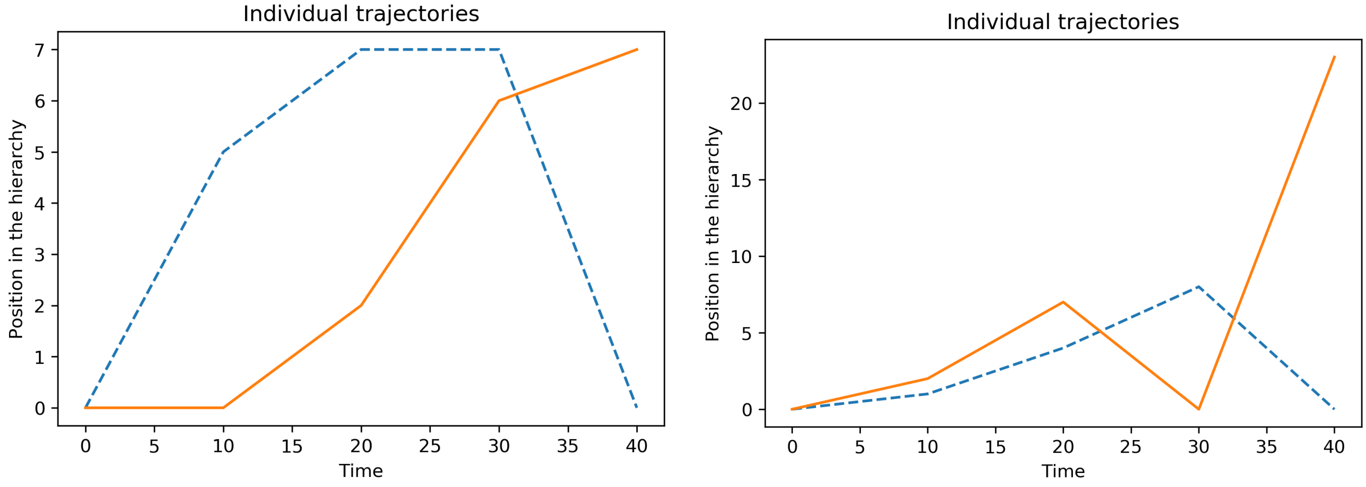

(a) Small, bounded group

(b) Big, bounded group

Figure S13: Trajectories for two different individuals in different groups and models: (a) group with $n = 8$ and (b) group with $n = 24$, and bounds.

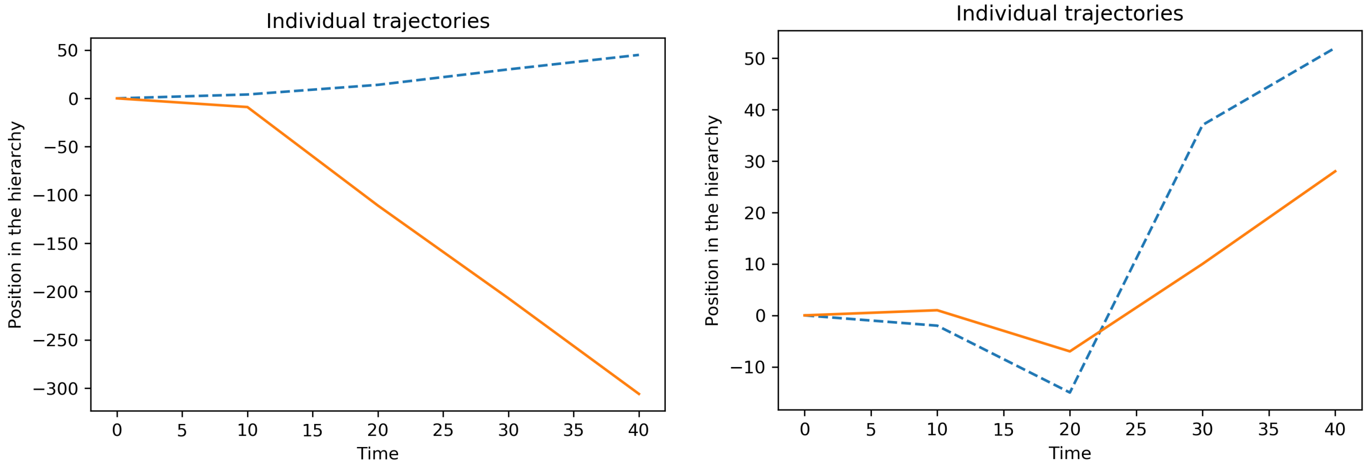

(a) Small, not bounded group

(b) Big, not bounded group

Figure S14: Trajectories for two different individuals in different groups and models: (a) group with $n = 8$ and (b) group with $n = 24$, and no bounds.

decreasing its rank, and in figure (b), we observe an initial perturbation before increasing their ranks. Thus, rank trajectories in unconstrained systems are steady. In Figure S13 (b) we observe initial movement in the trajectories before one of them quickly rose to the top of the hierarchy, but the difference with small models is that we do not observe as many individuals transitioning from the top to the bottom of the hierarchy as in models with smaller group size.

S3 Endogenously constrained model: Individual groups

Figures below show, for individual groups, the ranks distributions, payoff distributions per rank, and the relationship between fitness and ranks. Figure S15 shows the plots for small groups (size $n = 8$), and figure S16 shows the ones for big groups (size $n = 24$). Groups have been randomly selected, and the behavior is consistent between all of them.

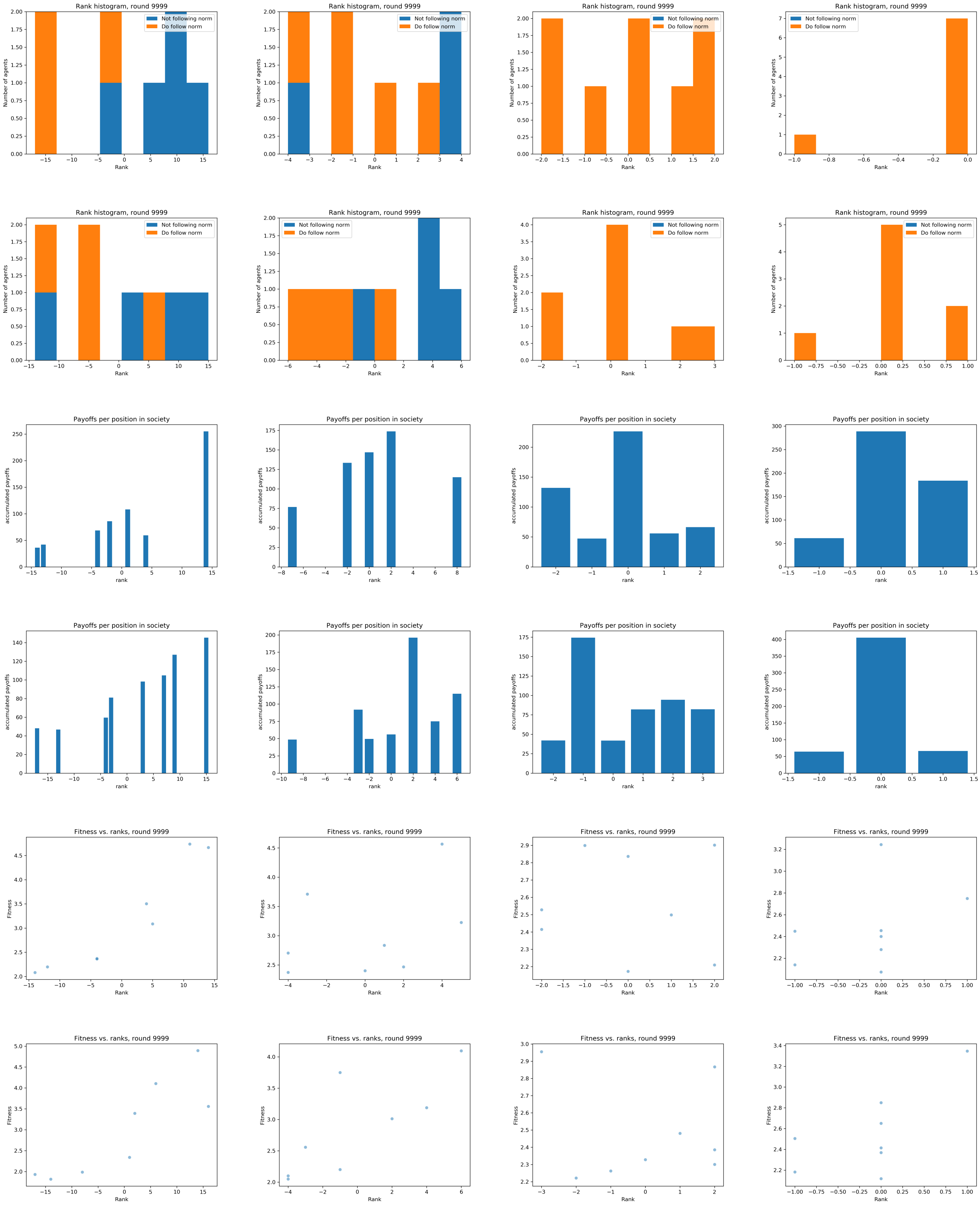

Figure S15: Simulation results for small groups, $n = 8$. Columns from left to right correspond to $\lambda = 0.1, 0.25, 0.5$ and 0.75. Top to bottom rows correspond to: Two rows of score histograms for two different groups; where blue indicates free riders—do not follow the norm of cooperating—and red indicates norm followers—cooperators. Two rows of payoff with respect the score plots. Two rows of fitness v. ranks plots.

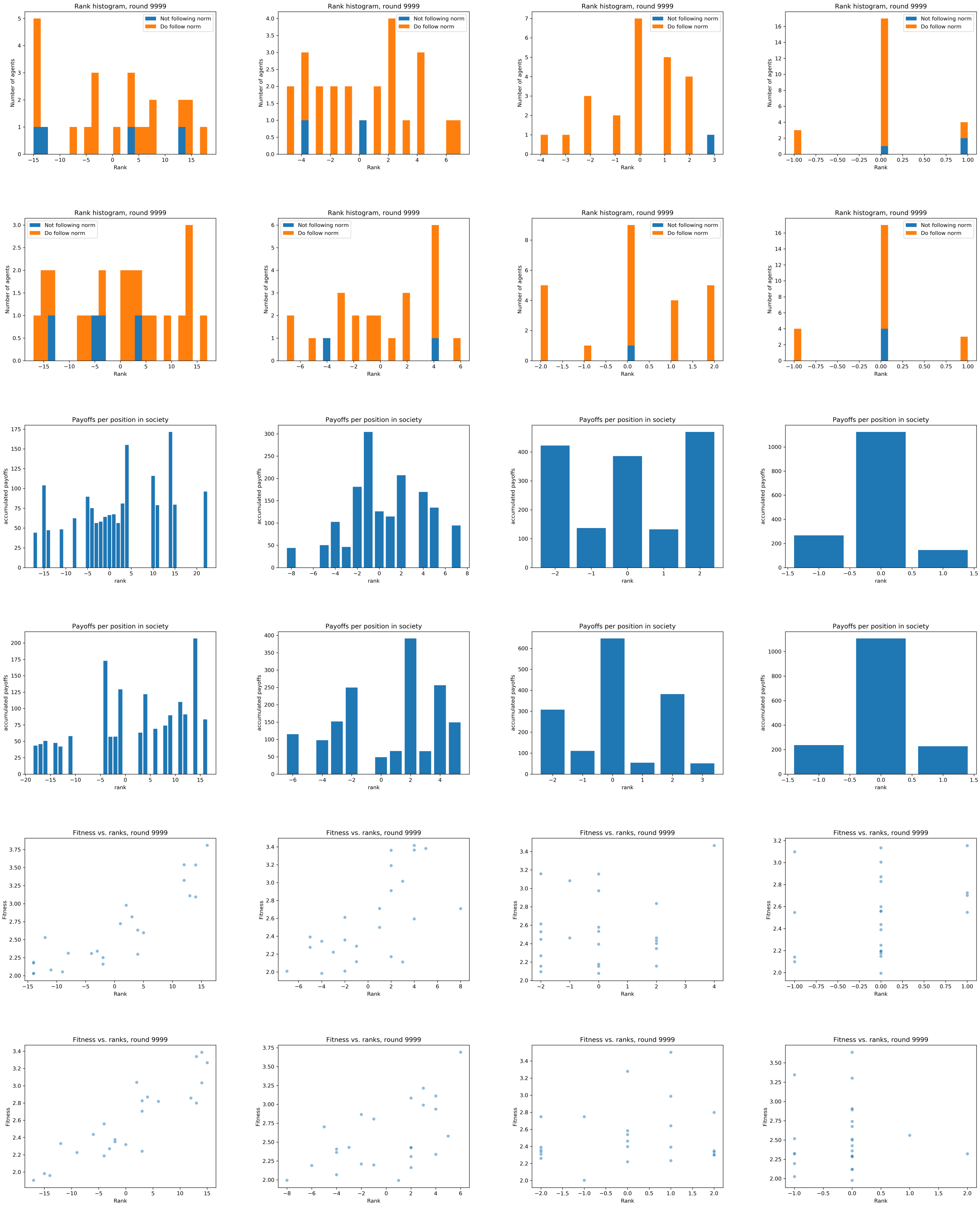

Figure S16: Simulation results for large groups, $n = 24$. Columns from left to right correspond to $\lambda = 0.1, 0.25, 0.5$ and 0.75. Top to bottom rows correspond to: Two rows of score histograms for two different groups; where blue indicates free riders—do not follow the norm of cooperating—and red indicates norm followers—cooperators. Two rows of payoff with respect the score plots. Two rows of fitness v. ranks plots.

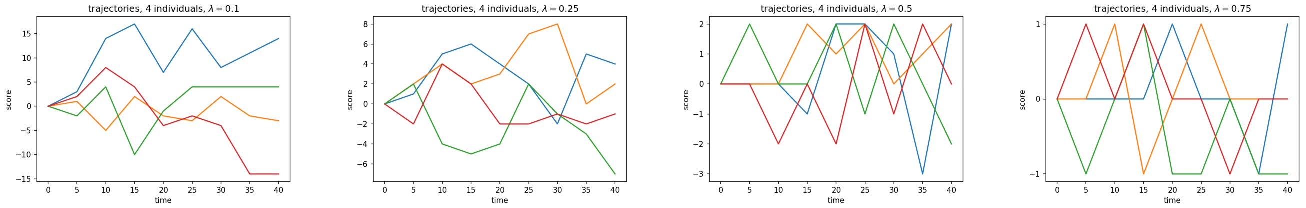

(a) $\lambda = 0.1$ (b) $\lambda = 0.25$ (c) $\lambda = 0.5$ (d) $\lambda = 0.75$

Figure S17: Trajectories for four different individuals within the same group for the endogenously constrained model. Groups consist of $n = 8$ individuals.

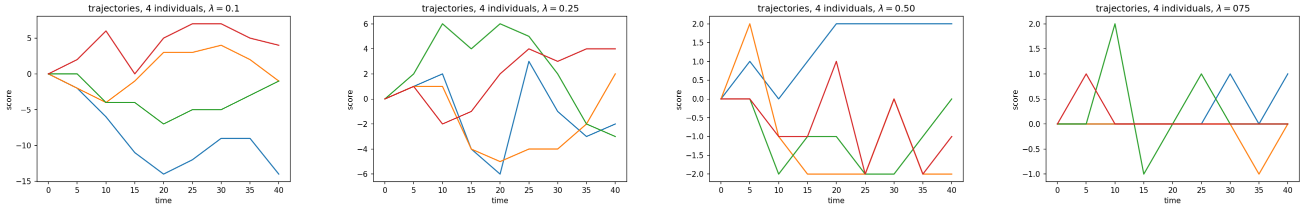

(a) $\lambda = 0.1$ (b) $\lambda = 0.25$ (c) $\lambda = 0.5$ (d) $\lambda = 0.75$

Figure S18: Trajectories for four different individuals within the same group for the endogenously constrained model. Groups consist of $n = 24$ individuals.
